# Supplementary material for: Tunneling current-controlled spin states in few-layer van der Waals magnets
Source: Nat Commun. 2024 May 1;15:3630. doi: 10.1038/s41467-024-47820-5 (PMC11063166; doi:10.1038/s41467-024-47820-5)
Supplement: Supplementary file 1 — Supplementary Information [file 41467_2024_47820_MOESM1_ESM.pdf]

**Supplementary Information:**

# Tunneling current-controlled spin states in few-layer van der Waals magnets

ZhuangEn Fu[1,2], Piumi I. Samarawickrama[1,2], John Ackerman[3], Yanglin Zhu[4], Zhiqiang Mao[4], Kenji Watanabe[5], Takashi Taniguchi[6], Wenyong Wang[1,2], Yuri Dahnovsky[1,2], Mingzhong Wu[7], TeYu Chien[1,2], Jinke Tang[1,2], Allan H. MacDonald[8], Hua Chen[9]* & Jifa Tian[1,2]*

1. Department of Physics and Astronomy, University of Wyoming, Laramie, Wyoming 82071, USA

2. Center for Quantum Information Science and Engineering, University of Wyoming, Laramie, Wyoming 82071, USA

3. Department of Chemical Biomedical Engineering, University of Wyoming, Laramie, Wyoming 82071, USA

4. Department of Physics, The Pennsylvania State University, University Park, Pennsylvania 16801, USA

5. Research Center for Electronic and Optical Materials, National Institute for Materials Science, 1-1 Namiki, Tsukuba 305-0044, Japan

6. Research Center for Materials Nanoarchitectonics, National Institute for Materials Science, 1-1 Namiki, Tsukuba 305-0044, Japan

7. Department of Physics and Department of Electrical and Computer Engineering, Northeastern University, Boston, Massachusetts 02115, USA

8. Department of Physics, The University of Texas at Austin, Austin, Texas 78712, USA

9. Department of Physics and School of Advanced Materials Discovery, Colorado State University, Fort Collins, Colorado 80523, USA

*e-mail: huachen@colostate.edu; jtian@uwyo.edu

## Supplemntary Note 1: Tunneling magnetoresistance in the linear response regime

To calculate the tunneling magnetoresistance for our model it is sufficient to consider the two insulator layers only. In this case we have

$$(\Sigma_D^{r,a})_{\tau\sigma\mathbf{k}}(\omega) = \mp i\Gamma \begin{pmatrix} 1 & \\ & 1 \end{pmatrix} \tag{1}$$

$$(\Sigma_D^{<})_{\tau\sigma\mathbf{k}}(\omega) = 2i\Gamma \begin{pmatrix} f_1(\hbar\omega) & \\ & f_2(\hbar\omega) \end{pmatrix}$$

$$(G_D^{r,a})_{\tau\sigma\mathbf{k}}(\omega) = \frac{1}{E_1^{\pm}E_2^{\pm}-t^2} \begin{pmatrix} E_2^{\pm} & t \\ t & E_1^{\pm} \end{pmatrix}$$

where $E_{1,2}^{\pm} = \hbar\omega - \epsilon_{\mathbf{k}}^{I} + \Delta\tau + J_{1,2}\sigma + \mu_I \pm i\Gamma$. It is then straightforward to get

$$(G_D^{<})_{\tau\sigma\mathbf{k}}(\omega) = \frac{2i\Gamma}{(E_1^{+}E_2^{+}-t^2)(E_1^{-}E_2^{-}-t^2)} \begin{pmatrix} |E_2|^2 f_1 + t^2 f_2 & tE_2^{+} f_1 + tE_1^{-} f_2 \\ tE_2^{-} f_1 + tE_1^{+} f_2 & |E_1|^2 f_2 + t^2 f_1 \end{pmatrix}. \tag{2}$$

In the present case the (electron number) current operator is

$$I_{\tau\sigma}(\mathbf{k}) = \frac{e}{i\hbar} \begin{pmatrix} 0 & -t \\ t & 0 \end{pmatrix} \tag{3}$$

The tunneling current for each spin and orbital is therefore

$$I_{\tau\sigma} = \mathrm{Im} \int_{-\infty}^{\infty} \frac{d\epsilon\prime}{2\pi} \int_{-\infty}^{\infty} N_I(\epsilon) d\epsilon \mathrm{Tr}[I_{\tau\sigma}(G_D^{<})_{\tau\sigma}(\epsilon', \epsilon)] \tag{4}$$

$$= \frac{et}{\hbar} \mathrm{Re} \int_{-\infty}^{\infty} \frac{d\epsilon\prime}{2\pi} \int_{-\infty}^{\infty} N_I(\epsilon) d\epsilon [(G_D^{<})_{21} - (G_D^{<})_{12}]$$

where $\epsilon' \equiv \hbar\omega$, $N_I(\epsilon)$ is the density of states in each insulator layer, and in the 2nd line we have omitted the $\tau\sigma$ subscripts. Moreover,

$$(G_D^{<})_{21} - (G_S^{<})_{12} = \frac{4\Gamma^2 t(f_1 - f_2)}{(E_1^{+}E_2^{+}-t^2)(E_1^{-}E_2^{-}-t^2)} \tag{5}$$

$$\approx \frac{4\Gamma^2 t}{|E_1|^2|E_2|^2} [\Theta(\mu_1 - \epsilon') - \Theta(\mu_2 - \epsilon')]$$

where we assumed $T = 0$ and $t$ is much smaller than the band gap. When $\mu_1 = \delta\mu > 0$ and $\mu_2 = 0$, Eq. 4 becomes

$$I_{\tau\sigma} \approx \frac{2et^2\Gamma^2 N_I}{\pi\hbar} \int_0^{\delta\mu} d\epsilon' \int_{-\frac{W}{2}}^{\frac{W}{2}} d\epsilon \frac{1}{|E_1|^2|E_2|^2} \tag{6}$$

where we have further assumed that the density of states $N_I(\epsilon) \approx N_I$ when $\epsilon \in [-\frac{W}{2}, \frac{W}{2}]$, $W$ being the band width, and 0 otherwise. Finally, if both $W$ and $\delta\mu$ are much smaller than the band gap, the integrand is approximately a constant. We thus obtain

$$I_{\tau\sigma} \approx \frac{2et^2\Gamma^2 N_I W\delta\mu}{\pi\hbar} \frac{1}{(\Delta\tau + J_1\sigma + \mu_I)^2(\Delta\tau + J_2\sigma + \mu_I)^2} \tag{7}$$

which means the tunneling conductance $G_{\tau\sigma} \equiv eI_{\tau\sigma}/\delta\mu$ is

$$G_{\tau\sigma} \approx \frac{e^2}{h}\frac{4t^2\Gamma^2 N_I W}{(\Delta\tau+J_1\sigma+\mu_I)^2(\Delta\tau+J_2\sigma+\mu_I)^2} \tag{8}$$

In the case of $CrI_3$ since the gap is between the two lowest bands having the same spin, the chemical potential $\mu_I = \frac{1}{2}(-J-\Delta-J+\Delta) = -J$. We can thus obtain, when the magnetizations of the two layers are parallel:

$$G_{\tau\sigma}^{p} \approx \frac{e^2}{h}\frac{4t^2\Gamma^2 N_I W}{(\Delta\tau+J\sigma-J)^4} \tag{9}$$

while when they are antiparallel

$$G_{\tau\sigma}^{ap} \approx \frac{e^2}{h}\frac{4t^2\Gamma^2 N_I W}{[(\Delta\tau-J)^2-J^2]^2}. \tag{10}$$

One can further obtain the tunneling magnetoresistance:

$$\mathrm{TMR} \equiv \frac{G^p-G^{ap}}{G^{ap}}. \tag{11}$$

For moderate values of $\Delta/J$, e.g. $\Delta/J = 0.7,0.8,0.9$, we find that $\mathrm{TMR} = 192\%, 109\%, 60\%$. We therefore chose $\Delta/J = 0.8$ to get the results in the main text so that the TMR is comparable to previous experimental results.

## Supplementary Note 2: Vanishing spin current in the antiferromagnetic state of the model without asymmetry

In this section we show that the tunneling spin current exactly vanishes in the antiferromagnetic state of our model when the two insulator layers are identical except for $J_1 = -J_2 = J$. This is most easily seen by using the standard Landauer formula applied to our model:

$$I_{\tau\sigma} = \frac{e}{\hbar}\int\frac{d\epsilon\prime}{2\pi}\int\frac{d^2\mathbf{k}}{(2\pi)^2}(f_1-f_2)4\Gamma^2\mathrm{Re}[(G_D^r)_{14}(G_D^a)_{41}] \tag{12}$$

where $G_D^{r,a}$ are given in Methods, and below we will omit the subscript $D$ for brevity. The tunneling currents obtained from this formula are consistent with that from the average inter-layer current discussed in Methods. This is a result of the fact that our model does not have other relaxation mechanisms except that through the lead self-energies and therefore the tunneling is fully coherent. When there is no asymmetry between the two insulator layers, in the antiferromagnetic state we have $(G_D^{r,a})_\uparrow$ being equivalent to $(G_D^{r,a})_\downarrow$ upon inverting the whole system:

$$(G_D^{r,a})_\uparrow = U^\dagger(G_D^{r,a})_\downarrow U,\ U_{ij} = \begin{pmatrix} 0 & 0 & 0 & 1 \\ 0 & 0 & 1 & 0 \\ 0 & 1 & 0 & 0 \\ 1 & 0 & 0 & 0 \end{pmatrix}_{ij} \equiv \delta_{i\bar{j}} \tag{13}$$

Therefore

$$(G_{D\uparrow}^{r,a})_{ij} = (U^\dagger G_{D\downarrow}^{r,a} U)_{ij} = (G_{D\downarrow}^{r,a})_{\bar{i}\bar{j}} = (G_{D\downarrow}^{r,a})_{\bar{j}\bar{i}} \tag{14}$$

where in the last step we have used $G_D = G_D^T$. Eq. 14 indicates that, for example, $(G_{D\uparrow}^{r,a})_{14} = (G_{D\downarrow}^{r,a})_{14}$, $(G_{D\uparrow}^{r,a})_{11} = (G_{D\downarrow}^{r,a})_{44}$. Using this result we can obtain, along with Eq. 12:

$$I_{\tau\uparrow} = I_{\tau\downarrow}. \tag{15}$$

Namely, the tunneling spin current exactly vanishes in the antiferromagnetic state. This result also suggests that as long as the time-reversal plus spatial inversion symmetry of the four-layer model in the antiferromagnetic state is broken, the tunneling spin current should generally be nonzero.

## Supplementary Note 3: Nonequilibrium spin accumulation and spin relaxation

Since the tunneling spin current transports up spin from upstream to downstream, which is balanced by local spin relaxation processes, naively we expect that (positive $I_s$ means the spin current flows from top to bottom)

$$\frac{d}{dt}\langle s_z\rangle_{\text{top}} = -I_s - \frac{\delta\langle s_z\rangle_{\text{top}}}{\tau_s} \tag{16}$$

$$\frac{d}{dt}\langle s_z\rangle_{\text{bottom}} = I_s - \frac{\delta\langle s_z\rangle_{\text{bottom}}}{\tau_s}$$

which means that in the steady state

$$\delta\langle s_z\rangle_{\text{top}} = -\delta\langle s_z\rangle_{\text{bottom}} = -I_s\tau_s. \tag{17}$$

Here $\tau_s$ is the spin relaxation time. Below we first show that the lead self-energies result in an effective $\tau_s$ and hence the spin accumulation in the present model.

The spin current for the present model is defined as

$$I_s = \frac{\hbar}{2e}\sum_\tau (I_{\tau\uparrow} - I_{\tau\downarrow}). \tag{18}$$

For convenience we use the Landauer formula Eq. 12 for $I_{\tau\sigma}$. Therefore

$$I_s = \frac{1}{2}\sum_{\tau\sigma}\int\frac{d\epsilon\prime}{2\pi}\int\frac{d^2\mathbf{k}}{(2\pi)^2}(f_1 - f_2)4\Gamma^2\text{Re}[(G_D^r)_{14}(G_D^a)_{41}]\sigma \tag{19}$$

We next directly calculate the nonequilibrium spin density. The spin densities in the top and bottom layers are

$$\langle s_z\rangle_{\text{top}} = \frac{\hbar}{2}\sum_{\tau\sigma}\int\frac{d\epsilon\prime}{2\pi}\int\frac{d^2\mathbf{k}}{(2\pi)^2}\text{Im}(G_D^<)_{11}\sigma \tag{20}$$

$$\langle s_z\rangle_{\text{bottom}} = \frac{\hbar}{2}\sum_{\tau\sigma}\int\frac{d\epsilon\prime}{2\pi}\int\frac{d^2\mathbf{k}}{(2\pi)^2}\text{Im}(G_D^<)_{44}\sigma$$

where the relevant matrix elements of $G_D^<$ can be further expressed as (below we drop the subscript $D$ for brevity)

$$(G^<)_{11} = (G^r\Sigma^<G^a)_{11} = (G^r)_{11}(\Sigma^<)_{11}(G^a)_{11} + (G^r)_{14}(\Sigma^<)_{44}(G^a)_{41} \tag{21}$$

$$= 2i\Gamma[f_1(G^r)_{11}(G^a)_{11} + f_2(G^r)_{14}(G^a)_{41}]$$
$$(G^<)_{44} = (G^r\Sigma^< G^a)_{44} = (G^r)_{41}(\Sigma^<)_{11}(G^a)_{14} + (G^r)_{44}(\Sigma^<)_{44}(G^a)_{44}$$
$$= 2i\Gamma[f_1(G^r)_{41}(G^a)_{14} + f_2(G^r)_{44}(G^a)_{44}]$$

However, the spin densities in the above expressions also include equilibrium contributions, since unlike the current, $\langle s_z \rangle$ does not vanish in equilibrium. Moreover, the local spin and charge densities can also change due to shifting the chemical potentials in all leads by the same amount, which is clearly an equilibrium effect. Such an equilibrium effect is also existent when $f_1 \neq f_2$ and is intertwined with the tunneling current induced nonequilibrium spin accumulation. Although generally speaking in a tunneling setup it may not always be possible to separate equilibrium and nonequilibrium contributions unambiguously for quantities that are nonzero in equilibrium, for the present model where there is only one metal layer attached to each lead it is sensible to define the nonequilibrium contributions as

$$\delta\langle s_z\rangle_{\text{top}}^{\text{neq}}(\mu_1,\mu_2) \equiv \langle s_z\rangle_{\text{top}}(\mu_1,\mu_2) - \langle s_z\rangle_{\text{top}}(\mu_1,\mu_1) \tag{22}$$
$$\delta\langle s_z\rangle_{\text{bottom}}^{\text{neq}}(\mu_1,\mu_2) \equiv \langle s_z\rangle_{\text{bottom}}(\mu_1,\mu_2) - \langle s_z\rangle_{\text{bottom}}(\mu_2,\mu_2).$$

This is equivalent to changing Eq. 21 into

$$(G^<)_{11}^{\text{neq}} = 2i\Gamma(f_2 - f_1)(G^r)_{14}(G^a)_{41} \tag{23}$$
$$(G^<)_{44}^{\text{neq}} = 2i\Gamma(f_1 - f_2)(G^r)_{41}(G^a)_{14}$$

As a result

$$\delta\langle s_z\rangle_{\text{top}}^{\text{neq}} = \frac{\hbar}{2}\sum_{\tau\sigma}\int\frac{d\epsilon\prime}{2\pi}\int\frac{d^2\mathbf{k}}{(2\pi)^2}2\Gamma(f_2 - f_1)\text{Re}[(G^r)_{14}(G^a)_{41}]\sigma \tag{24}$$
$$\delta\langle s_z\rangle_{\text{bottom}}^{\text{neq}} = \frac{\hbar}{2}\sum_{\tau\sigma}\int\frac{d\epsilon\prime}{2\pi}\int\frac{d^2\mathbf{k}}{(2\pi)^2}2\Gamma(f_1 - f_2)\text{Re}[(G^r)_{41}(G^a)_{14}]\sigma$$

Comparing Eqs. 24 and 19, we obtain

$$\delta\langle s_z\rangle_{\text{top}}^{\text{neq}} = -\delta\langle s_z\rangle_{\text{bottom}}^{\text{neq}} = -I_s\frac{\hbar}{2\Gamma} \tag{25}$$

Therefore the leads self-energies correspond to a spin-relaxation time

$$\tau_s^{\text{model}} = \frac{\hbar}{2\Gamma}. \tag{26}$$

Because spin-relaxation in our model occures in the leads, $\tau_s^{\text{model}}$ here is equal to the escape time from the device and therefore related to the contact resistance. In a more realistic model which incorporates spin-relaxation within the device, these two quantities can vary independently.

Based on the above discussion, we calculate the nonequilibrium spin accumulation in the top and bottom metal layers in this work by first using Eq. 24 and then rescaling them by $\tau_s^{\text{exp}}/\tau_s^{\text{model}}$, with $\tau_s^{\text{exp}}$ (or simply $\tau_s$ used in the main text) the experimentally determined spin relaxation time in graphene.

Including spin relaxation explicitly in the NEGF calculations through, e.g., a spin non-conserving self-energy [1], will be left for a future study.

## Supplementary Note 4: Spin accumulation in the SAP state with different types of asymmetry between the two $CrI_3$ layers

Besides different sizes of the local exchange splitting in the two insulator layers, other types of asymmetry which should generally coexist in the real system can also contribute to the nonzero tunneling spin current in the antiferromagnetic state, according to the discussion in Supplementary Note 2. Figure S18 includes the results by considering other types of symmetry breaking mechanisms. Figure S18a and S18b correspond to the case of the two insulator layers having different orbital splittings. Since the top layer has a smaller Δ, the tunneling electrons see a smaller barrier when crossing it and therefore carry the spin polarization set by this layer. Figure S18c and S18d are obtained by making the chemical potential $\mu_I$ of the bottom insulator layer slightly higher than that of the top layer, mimicking the effect of an out-of-plane electric field. As a result the tunneling spin current becomes nonzero and more importantly has a pronounced asymmetry between positive and negative biases. Finally panels e and f show the results of different hopping amplitudes between the two insulator layers and their respective neighboring metal layers. In realistic samples these symmetry-breaking mechanisms should in principle coexist and contribute to the net spin current and spin accumulation. Their signs and sizes are however determined by microscopic details of the experimental samples which are beyond the present model. We focused on the asymmetric exchange splitting in the main text since in comparison with the mechanisms discussed in this section the former has a more definite correlation with other experimental facts and is less ambiguous.

## Supplementary Note 5: "Anomoly" of the magnetic switching

It appears in the rightmost plot of panel (b) of the Fig. S6 that in the negative bias branch there is an SAP to SP as we sweep to negative biases at 0.534 T, which seems to contradict the unidirectional rule we proposed. However, we think that there are at least two possible explanations within our theoretical framework.

1. As illustrated in Fig. 4d of the main text, SAP to SP switching under increasing current density occurs only for positive bias *if the magnetization direction of the bottom layer is pinned by the magnetic field*. If the top layer has a larger magnetization and is pinned by the magnetic field, the opposite will happen. While we expect the graphene/$CrI_3$/graphene device prepared by mechanical exfoliation and transfer to be sufficiently uniform in the ~0.5μm$^2$ tunnel junction area, we cannot exclude the possibility that there exist certain regions with a flipped layer/magnetization locking.

2. The stochastic and unidirectional switching events occur ideally in different ranges of current densities. At high current densities significant heating leads to deviation of the dynamic magnetic states in the bilayer from the static SAP and SP states. In such a case conventional spin-transfer torque is effective and indeed the SP state always seems to be favored over the SAP state at the high-current end of the stochastic switching regions (see e.g., Fig. 3a in the main text). However, different domains in general have different high- and low-bias ranges thus defined, and the "anomalous" SAP to SP switching at negative bias can originate from the low-bias end of the stochastic switching range of a different domain. In addition, we also found a similar SAP to SP switching feature in the negative-bias range between 0.58 T and 0.575 T in Fig. S6a, which as the magnetic field keeps decreasing merges into the stochastic switching region at around 0.575 T.

## Supplementary Note 6: Comparison of coercivities for current-induced switching events in a 2L $CrI_3$

We have made a direct comparison of coercivities for the current-induced switching between the B = 0.514 T and 0.564 T in Fig. S16a. We focus on the hysteresis loops in the positive bias side. We notice that the tunneling current induced hysteresis loop starts around 0.514 T, suggesting the corresponding magnetic domains are in SAP state before current (blue curve) induced switching. The hysteresis loop disappears at 0.564 T, suggesting the related coercivity of this magnetic domain is around 0.05 T. Then, we look at the current induced switching of the same domain at 0.514 T as shown in Fig. S16b. We extract the tunneling current difference ($\Delta I$) of the hysteresis loop by $I_{high}$ - $I_{low}$ = 1843 nA, which refers to the current coercivity of this magnetic domain. We further plotted the magnetic field dependence of $I_{high}$, $I_{low}$ and $\Delta I$ in Fig. S16c. We see that, as expected, the corresponding current coercivity decreases with the increasing magnetic field.

## Supplementary Note 7: Reproducibility of unidirectional magentic switching and stochastic switching in $CrI_3$ few layers

The unidirectional switching behavior is highly reproducible within the same device during a long period of measurement time as evidenced by Figs. S6, S9-S11. Most importantly, the unidirectional hysteresis has also been observed in other devices with different thicknesses. For example, in addition to the results taken from the 2L and 4L $CrI_3$ devices presented in the main text, we have demonstrated the same unidirectional magnetization switching and stochastic switching in a 5L $CrI_3$ device, as shown in Fig. S17. We confirmed that the observed unidirectional magnetization reversal and stochastic switching can be observed in all of our working devices with different thicknesses that we have measured thus far (see Table. S2). We refer

the working devices as the graphene/$CrI_3$/graphene tunneling junctions with nonlinear I-V characteristics (characteristics of the tunneling behavior) and clear magnetic field dependent tunneling magnetoresistance.

## Supplementary Note 8: Estimate the switching current density of the unidirectional spin-state transition

We calculated the switching current density by dividing the tunneling current by the estimated area of a single domain in $CrI_3$ by directly analyzing the transport data in Fig. 2a. To extract the area of the magnetic domain and the current responsible for the spin state transition, we assume the Ohm's law is valid, and the resistances of the magnetic domains with SP and SAP states connected in parallel in the circuit as shown in Fig. S5. Figure S5a-c depicts the simplified circuits of the 2L $CrI_3$ tunnel junction device displaying a layer SP state, a layer SAP state, and two magnetic domains with both SP and SAP states, respectively. As demonstrated in Fig. 2b of the main text, near the SAP to SP transition region, the layer magnetic state of the 2L $CrI_3$ is SAP when the magnetic field is below 0.2 T and shifts to SP when it surpasses 0.7 T. Furthermore, Figure 2b reveals that the current-induced transition from the SAP state (marked as ①) to the SP state (marked as ②) can be interpreted as the SAP state (with two magnetic domains in the layer) flipping to SP (with only one magnetic domain now). It's worth mentioning that there may be one or two additional small domains. However, their influence on the resistance change is minimal compared to the SAP to SP transition highlighted in the inset of Fig. 2a of the main text. Now, we can use the data in Fig. S5d to estimate the area of the magnetic domain with the SAP state. When the magnetic field is above 0.7 T, the layer magnetic state is SP, and we have $R_{SP} = \frac{\rho_{SP} L}{A}$, where $R_{SP}$ is the resistance of the junction, $A$ is the junction area (~ 0.5 μm$^2$), $L$ is the thickness of the sample, and $\rho_{SP}$ is the corresponding resistivity. At 0.7 T (see Fig. S5d), we have $\rho_{SP} L = R_{SP} A = 0.5490\ M\Omega \times\ 0.5\ \mu m^2 = 0.2745\ M\Omega \cdot \mu m^2$. Similarly, at 0.2 T (Fig. S5d), the layer magnetic state is SAP, and we have $R_{SAP} = \frac{\rho_{SAP} L}{A}$, where $R_{SAP}$ and $\rho_{SAP}$ are the resistance and resistivity of the tunnel junction, respectively. Then, $\rho_{SAP} L = R_{SAP} S_{SAP} = 0.603\ M\Omega \times\ 0.5\ \mu m^2 = 0.3015\ M\Omega \cdot \mu m^2$. In the case of the intermediate state with one SP domain and one SAP domain (Fig. S5c) in the 2L $CrI_3$, the total resistance $R'$ of the intermediate state is $\frac{1}{R'} = \frac{1}{R'_{SP}} + \frac{1}{R'_{SAP}}$, where $R'_{SP}$ and $R'_{SAP}$ are the resistances of the SP and SAP domains, respectively. Then, we have $\frac{1}{R'} = \frac{1}{\frac{\rho_{SP} L}{A - A_{SAP}}} + \frac{1}{\frac{\rho_{SAP} L}{A_{SAP}}}$, where $A_{SAP}$ is the area of the domain with an SAP state. From Fig. S5d, we know $R' = 0.573$ MΩ, resulting in the area ($A_{SAP}$) of the magnetic domain with an SAP state of ~0.235 μm. From Fig. S5d, we know at state ①, $R' = 0.573$ MΩ, resulting in the area ($A_{SAP}$) of the magnetic domain with an SAP state is ~0.235 μm$^2$. At state ② and $R' = 0.556$ MΩ, the estimated area of the magnetic domain is ~ 0.072

μm$^2$. Thus, the area of the magnetic domain that got switched from ① to ② is ~ 0.163 μm$^2$, which is comparable with the areas of the magnetic domains of $CrI_3$ thin layers captured by single-spin microscopy, as referenced in Ref. 35. Next, we calculate the switching current, $I_{SAP}$, which is responsible for the SAP to SP transition at the bias current $I$ of 3 μA. From Fig. S5d, we have $I_{SAP} = \frac{IR'_{SP}}{R'_{SP}+R'_{SAP}} = 1.18$ μA. Thus, the switching current density ($\frac{I_{SAP}}{A_{SAP}}$) is ~724 A/cm$^2$, being around three orders of magnitude lower than the values reported in previous studies employing SOT. We note that the transition between SAP and SP states can be realized at a much lower current of ~100 nA, and the switching current density could be even lower.

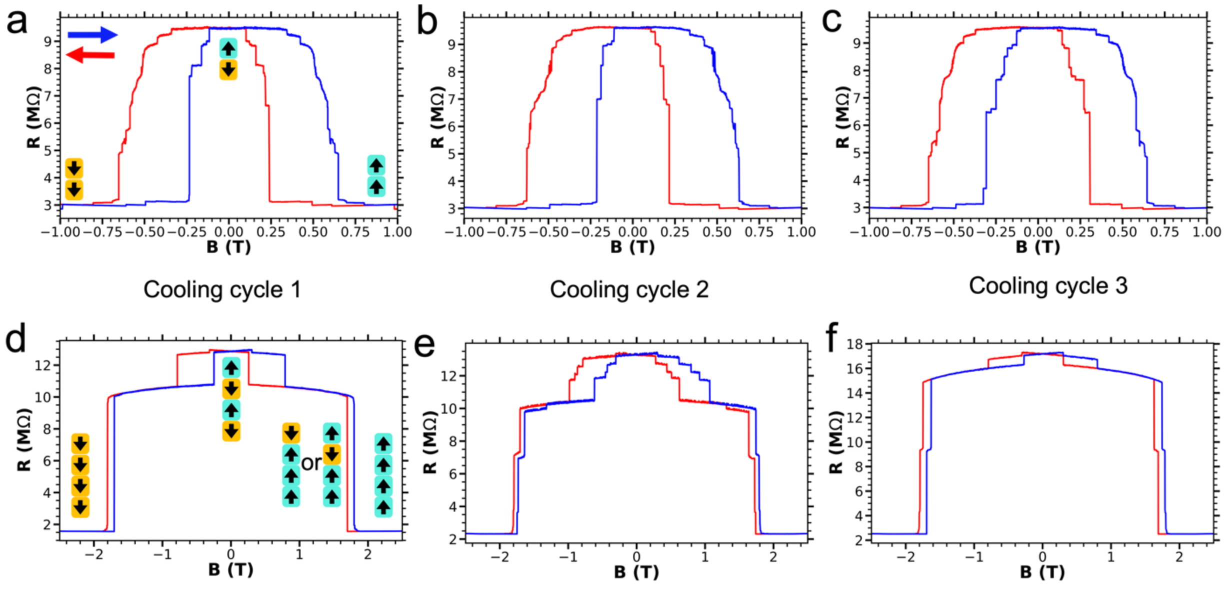


**Fig. S1| Cooling history-dependent magnetic domain formation in 2L and 4L $CrI_3$.** Tunneling resistance as a function of an applied magnetic field with different cooling cycles for **a-c** a 2L $CrI_3$ tunnel junction device and **d-f** a 4L $CrI_3$ tunnel junction device. All measurements were performed at $T$ = 1.5 K. One cooling cycle represents warming up the device to room temperature and cooling it down to the base temperature again.

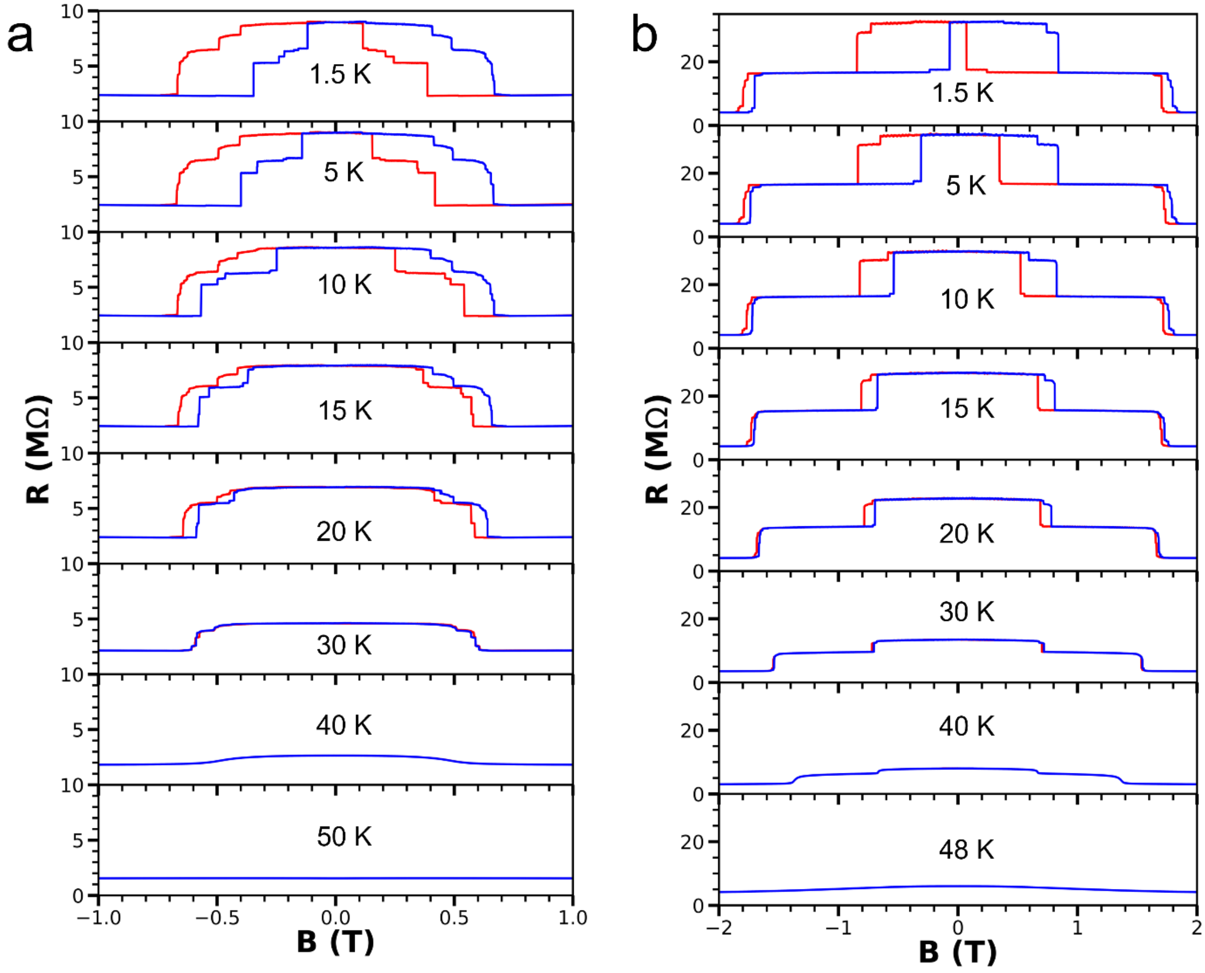


**Fig. S2| Temperature dependence of tunneling magnetoresistance for 2L and 4L $CrI_3$.** Tunneling resistance as a function of an applied out-of-plane magnetic field at different temperatures at a constant bias of 350 mV for **a** a 2L $CrI_3$ and 300 mV for **b** a 4L $CrI_3$, respectively.

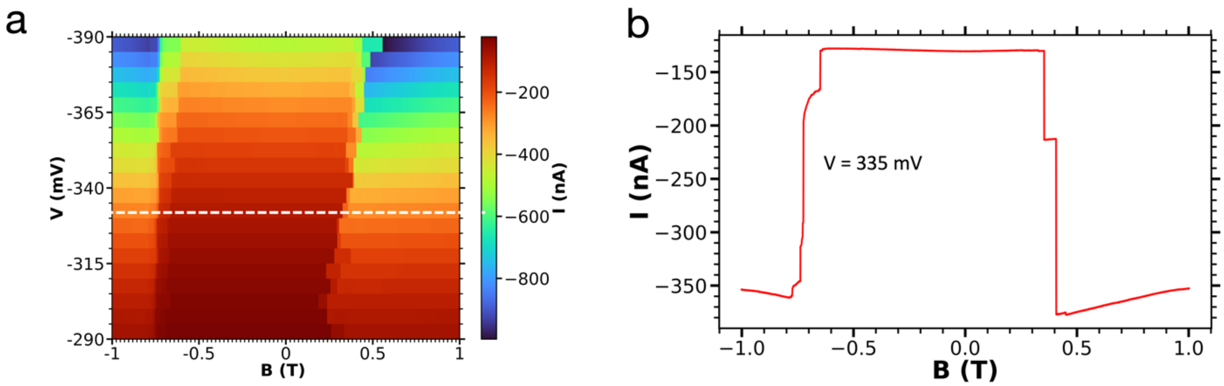


**Fig. S3| Bias voltage-dependent coercivity of a 2L $CrI_3$ (Device 2). a** Tunneling current as a function of both bias voltage and applied magnetic field of a 2L $CrI_3$, where the magnetic field was swept from -1 to 1 T. **b** Selected line profile from the white-dashed line indicated in (a). The measurements were performed at $T$ = 1.5 K.

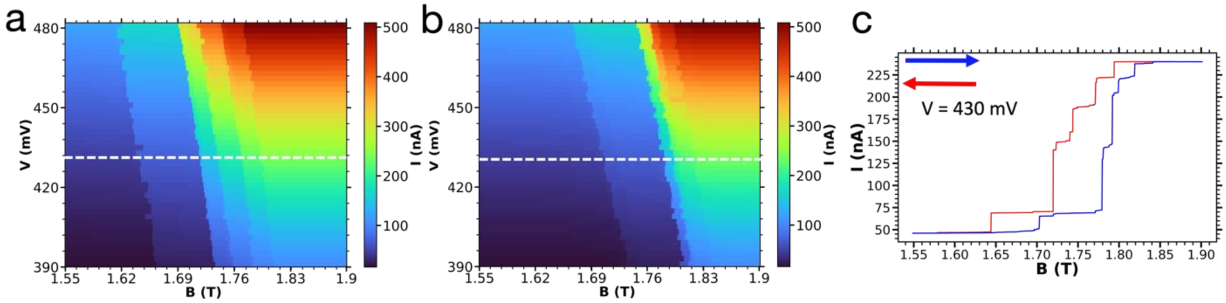


**Fig. S4| Bias voltage-dependent coercivity of a 4L $CrI_3$. a** and **b** Tunneling current as a function of both bias voltage and magnetic field of the 4L $CrI_3$, where B was swept from 1.9 to 1.55 T (a) and from 1.55 to 1.9 T (b). **c** Selected line profiles from the white-dashed line indicated in (**a**) and (**b**). The measurements were performed at $T$ = 1.5 K.

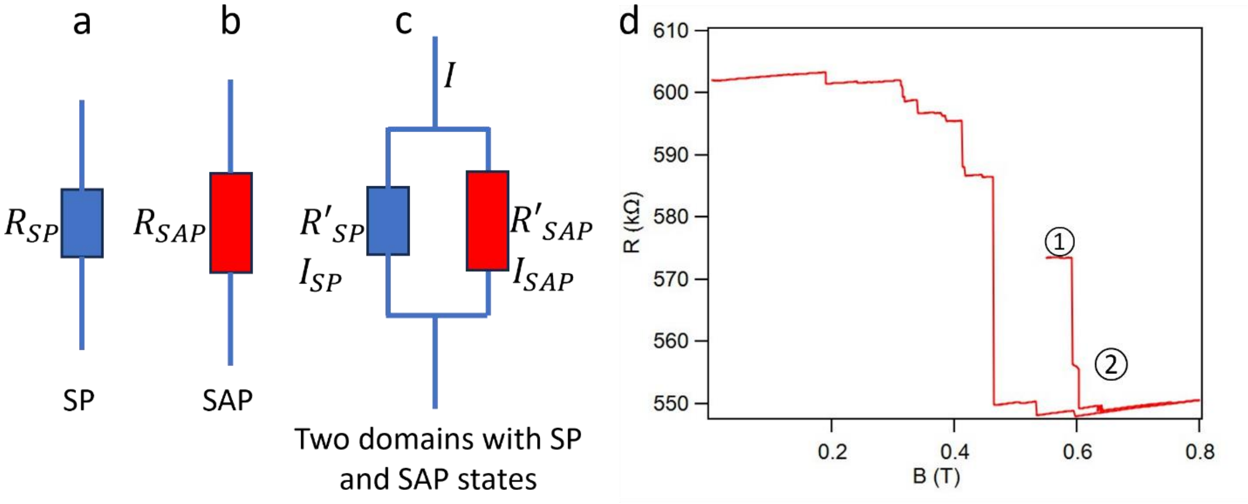


**Fig. S5| Simplified circuits for estimating the size for magnetic domain.** The circuits for 2L $CrI_3$ tunnel device with **a** layer SP state, **b** layer SAP state, and **c** two magnetic domains with both SP and SAP states. **d** Tunneling resistance as a function of the magnetic field at a bias current of 1 μA (top panel of Fig. 2a).

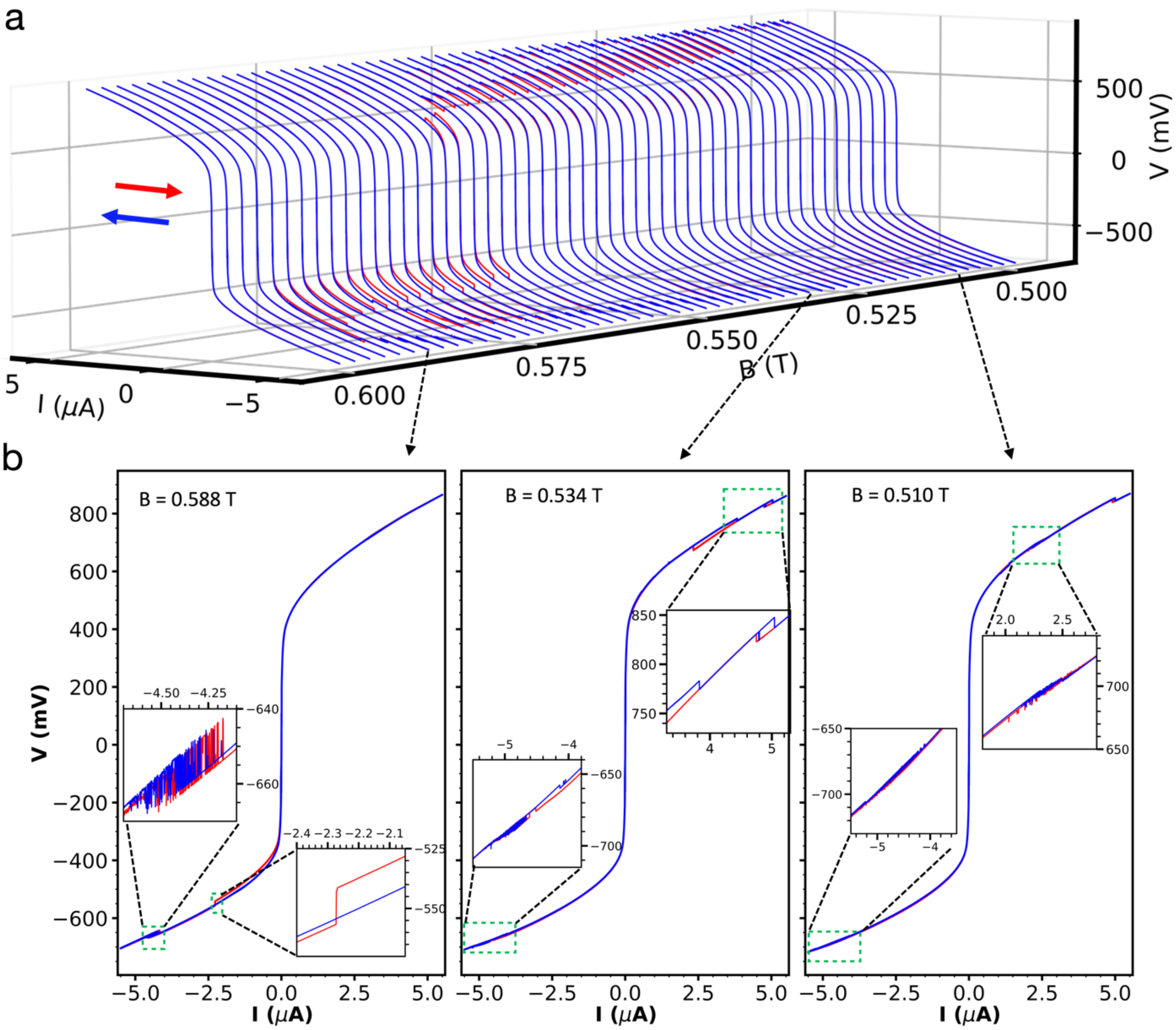


**Fig. S6| Magnetic field dependent *I*-*V* characteristics for a 2L $CrI_3$ at $T$ = 1.5 K. a** Voltage as a function of applied current at different magnetic fields ranging from 0.5 to 0.6 T with a step size of 2 mT. **b** Representative I-V curves measured at magnetic fields of 0.588, 0.534, and 0.510 T, respectively. Insets are zoom-in curves from the corresponding dashed green boxes.

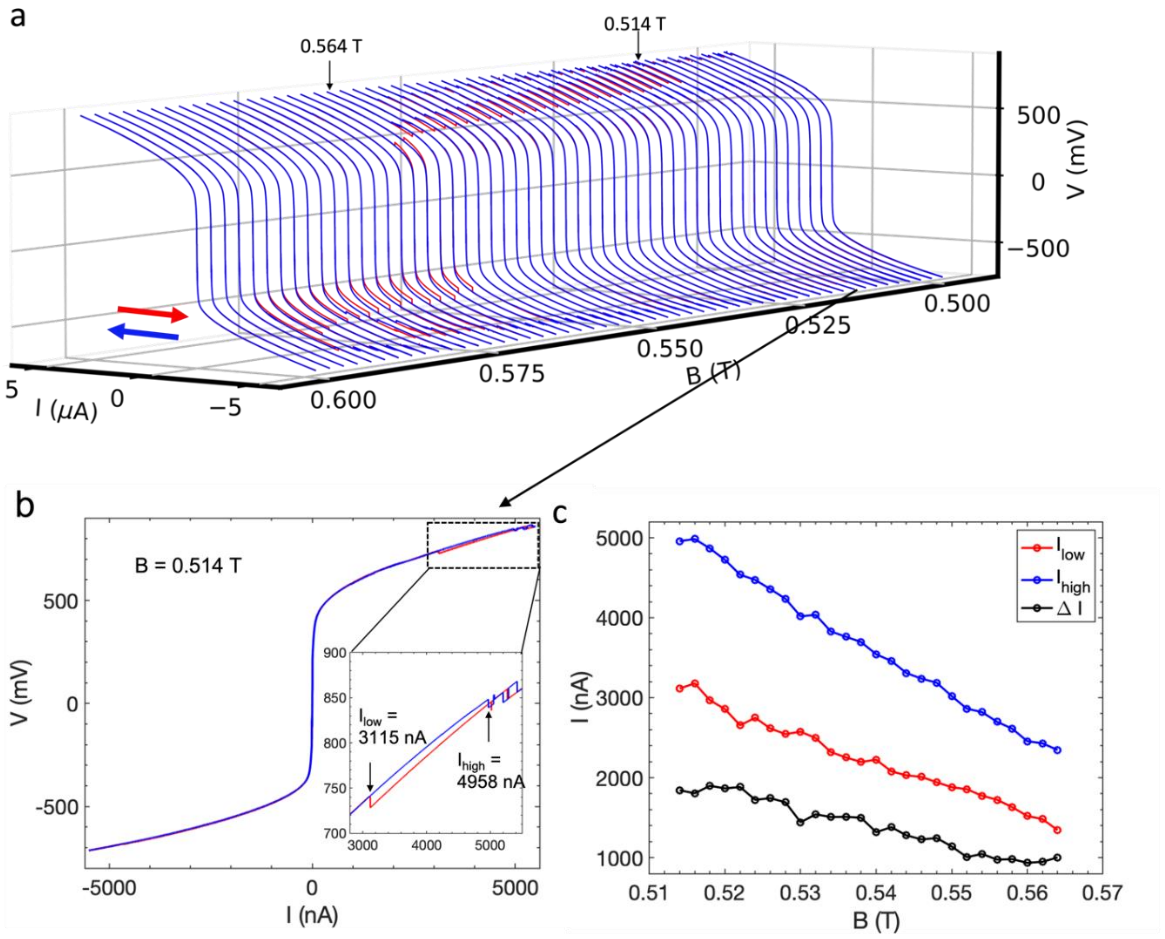


**Fig. S7| Magnetic field dependent *I-V* characteristics and current coercivity for a 2L $CrI_3$. a** Voltage as a function of applied current at different magnetic fields ranging from 0.5 to 0.6 T with a step size of 2 mT. **b** Representative *I-V* curves measured at magnetic fields of 0.514T. Inset is zoom-in curves from the dashed black box. **c** B-field-dependence of the current coercivity for the hysteresis loop around 3000 nA, as shown in the inset of (**b**). The $I_{low}$ and $I_{high}$ are the current of the red and blue curve at the transition points, respectively. $\Delta I = I_{high} - I_{low}$. All the data were measured at 1.5 K.

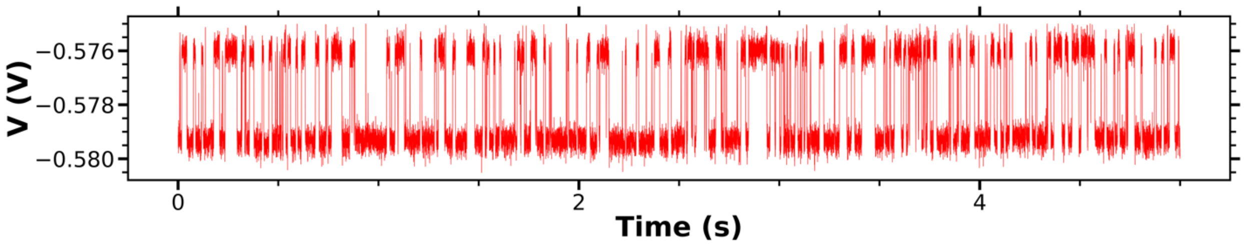


**Fig. S8|** Time snapshot of voltage for an applied DC current in the fluctuation region with a time scale of 5 s of a 2L $CrI_3$. The measurement was performed at $B$ = 0.550 T and $T$ = 1.5 K.

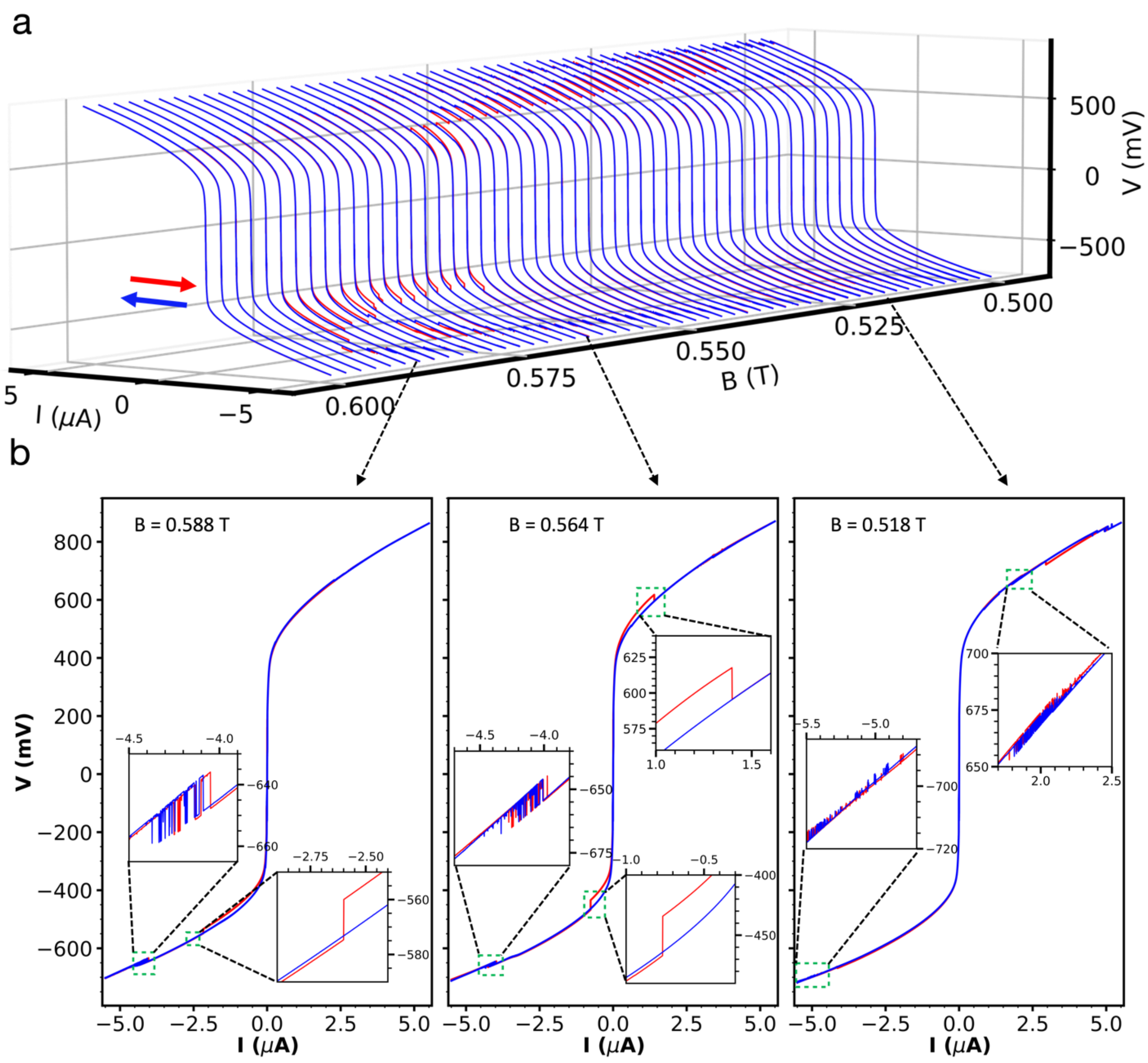


**Fig. S9| Magnetic field dependent *I-V* characteristics for a 2L $CrI_3$ at $T$ = 10 K. a** Voltage as a function of applied current at different magnetic fields ranging from 0.5 to 0.6 T with a step size of 2 mT. **b** Representative I-V curves measured at magnetic fields of 0.588, 0.564, and 0.518 T, respectively. Insets are zoom-in curves from the corresponding dashed green boxes.

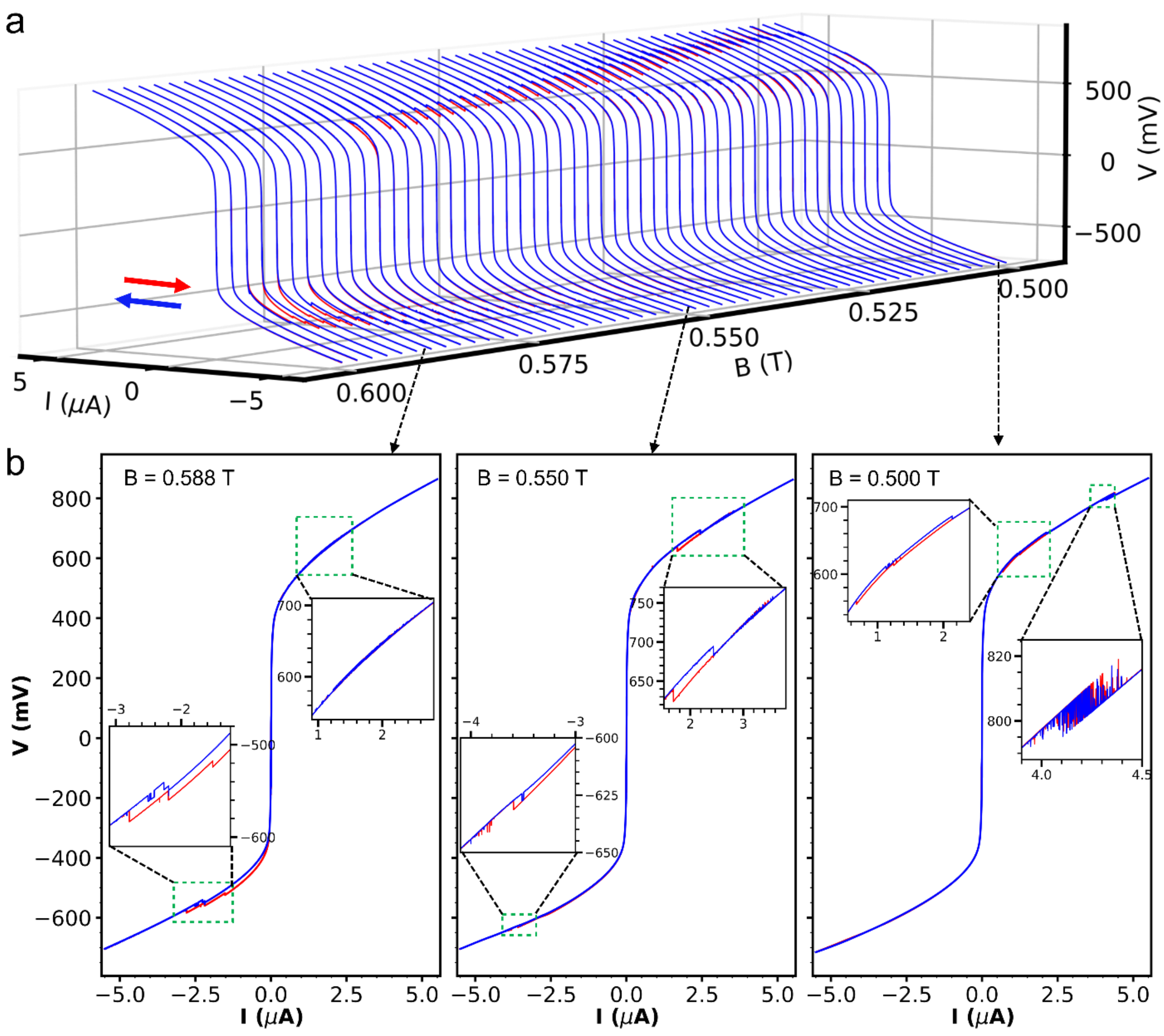


**Fig. S10| Magnetic field dependent *I*-*V* characteristics for a 2L $CrI_3$ at $T$ = 20 K. a** Voltage as a function of applied current at different magnetic fields ranging from 0.5 to 0.6 T with a step size of 2 mT. **b** Representative *I*-*V* curves measured at magnetic fields of 0.588, 0.550, and 0.500 T, respectively. Insets are zoom-in curves from the corresponding dashed green boxes.

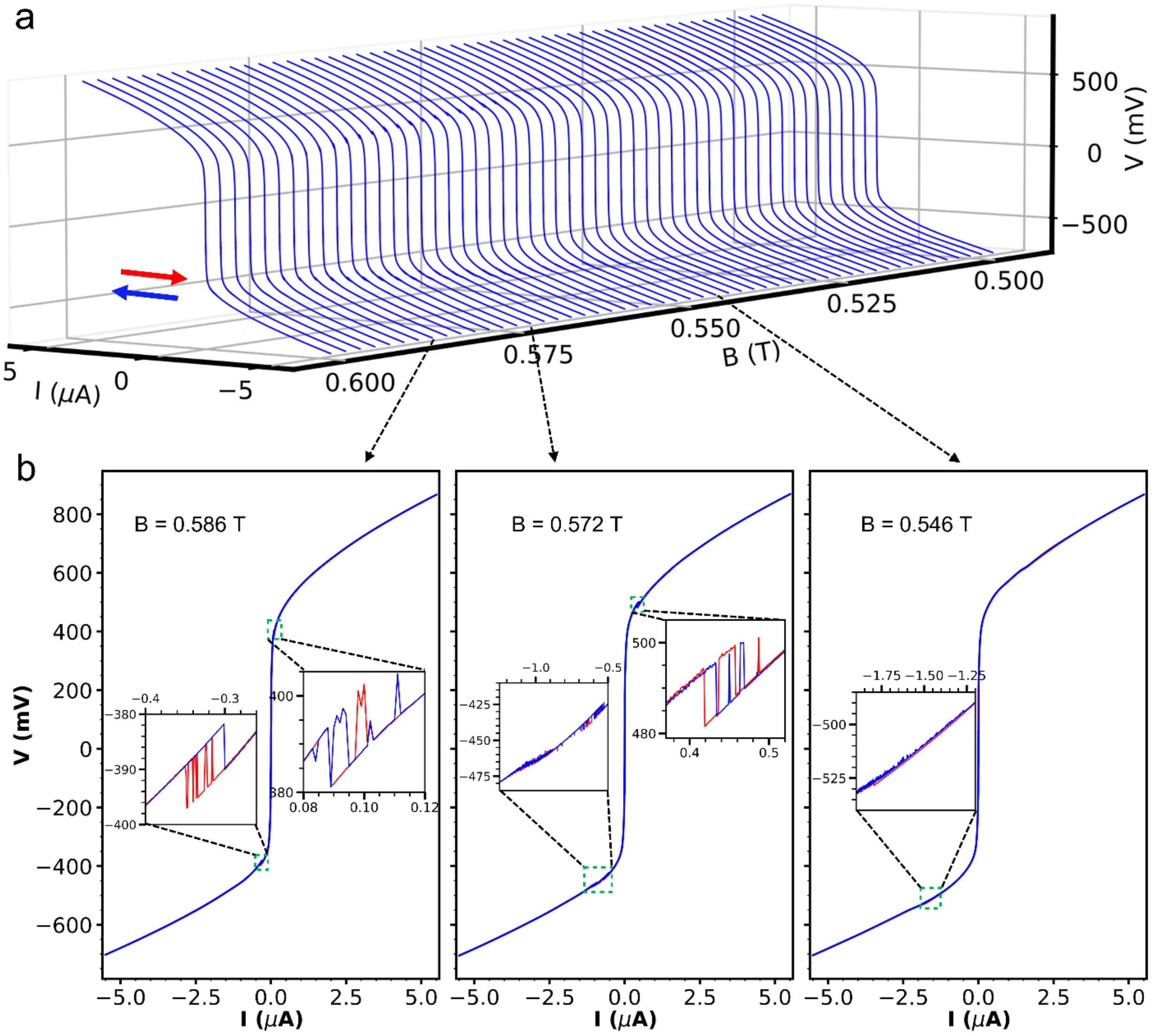


**Fig. S11| Magnetic field dependent *I-V* characteristics for a 2L $CrI_3$ at $T$ = 30 K. a** Voltage as a function of applied current at different magnetic fields ranging from 0.5 to 0.6 T with a step size of 2 mT. **b** Representative *I-V* curves measured at magnetic fields of 0.586, 0.572, and 0.546 T, respectively. Insets are zoom-in curves from the corresponding dashed green boxes.

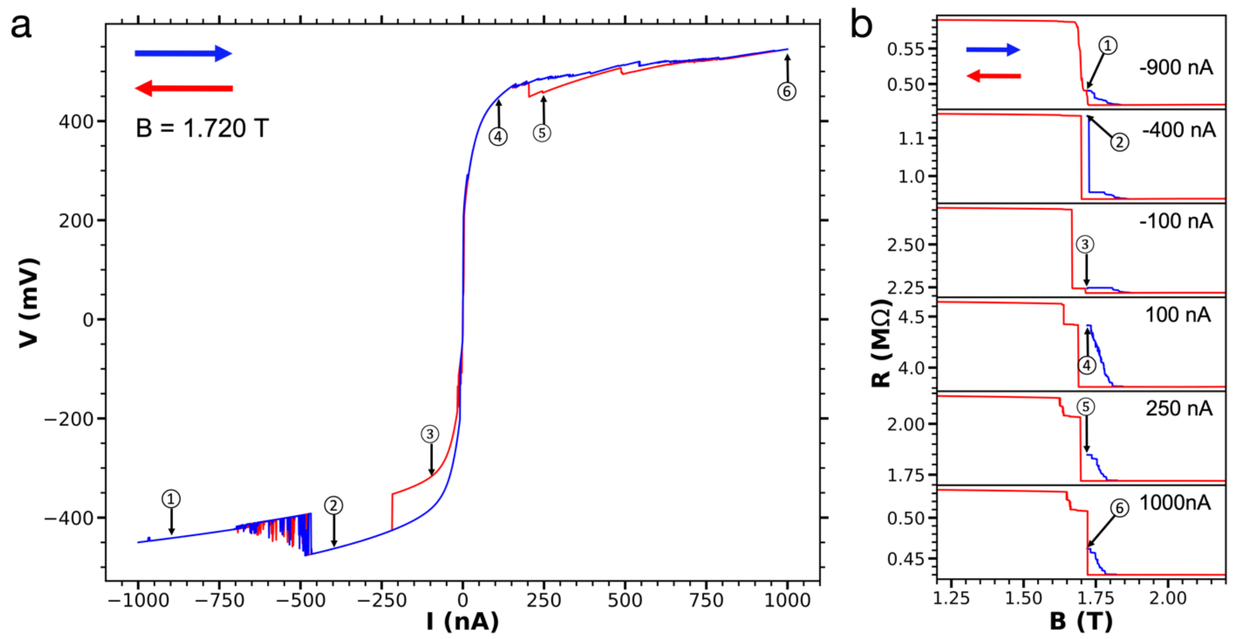


**Fig. S12| Current-driven magnetization reversal in a 4L $CrI_3$. a** Voltage as a function of applied current of a 4L $CrI_3$ measured at $B$ = 1.720 T. **b** Tunneling resistance as a function of an applied out-of-plane magnetic field at different bias currents. The initial magnetic states are prepared at $B$ = 1.720 T, then the current is ramped to 1 μA and then back to the target currents as indicated in each graph. The circled numbers in (**a**) and (**b**) indicate the corresponding initial spin states. All the measurements were performed at $T$ = 1.5 K.

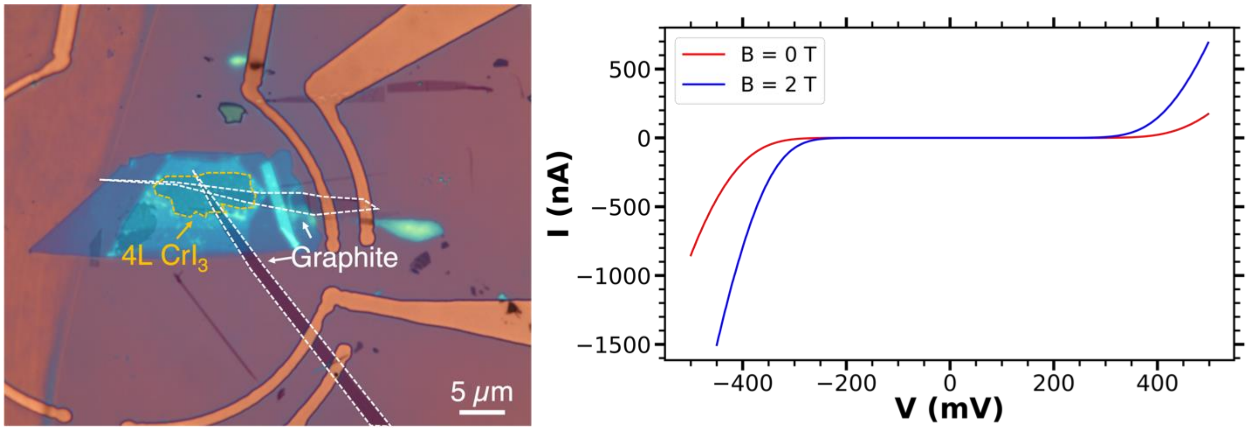


**Fig. S13| *I-V* characteristics of a 4L $CrI_3$ tunneling device. a** Optical image of a 4L $CrI_3$ tunneling device. **b** *I-V* characteristics of the 4L $CrI_3$ tunneling device measured at $B = 0$ and 2 T. The measurements were performed at $T = 1.5$ K.

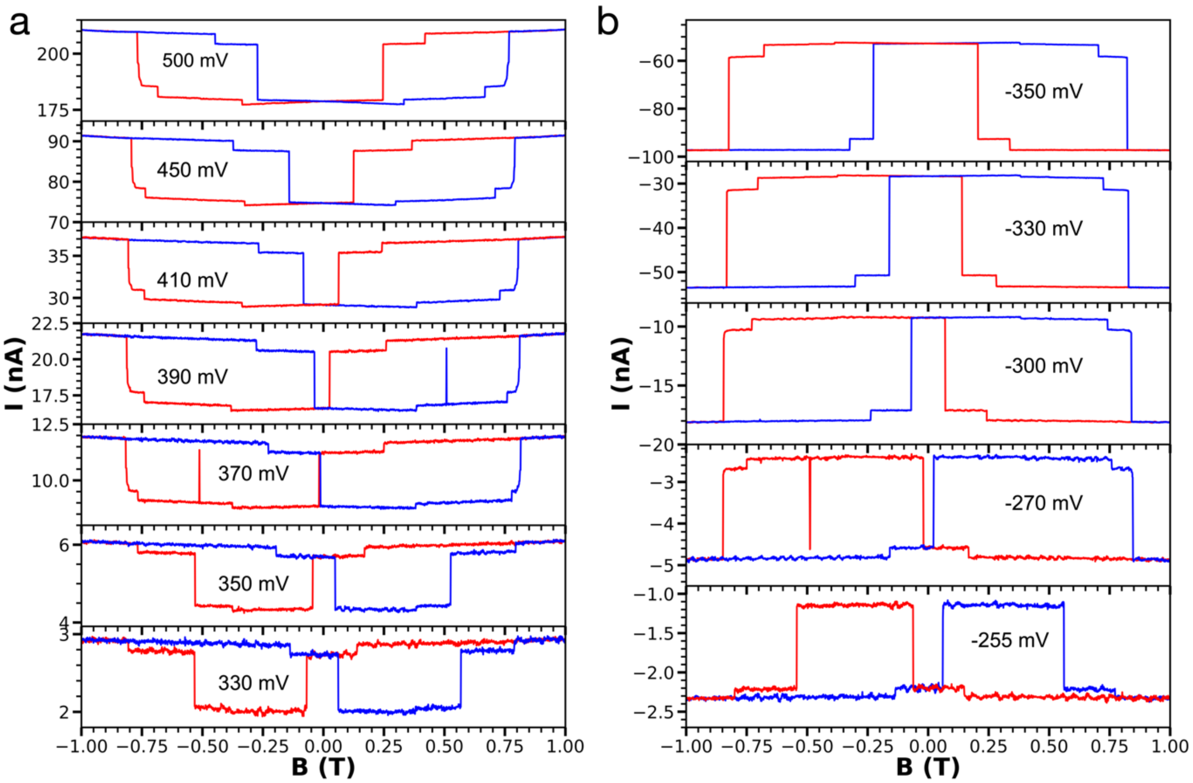


**Fig. S14| Bias voltage-dependent coercivity in a 4L $CrI_3$. a** and **b** Tunneling current as a function of magnetic field of the 4L $CrI_3$ tunneling device measured at both positive (**a**) and negative (**b**) bias voltages. All the measurements were carried out at $T = 1.5$ K.

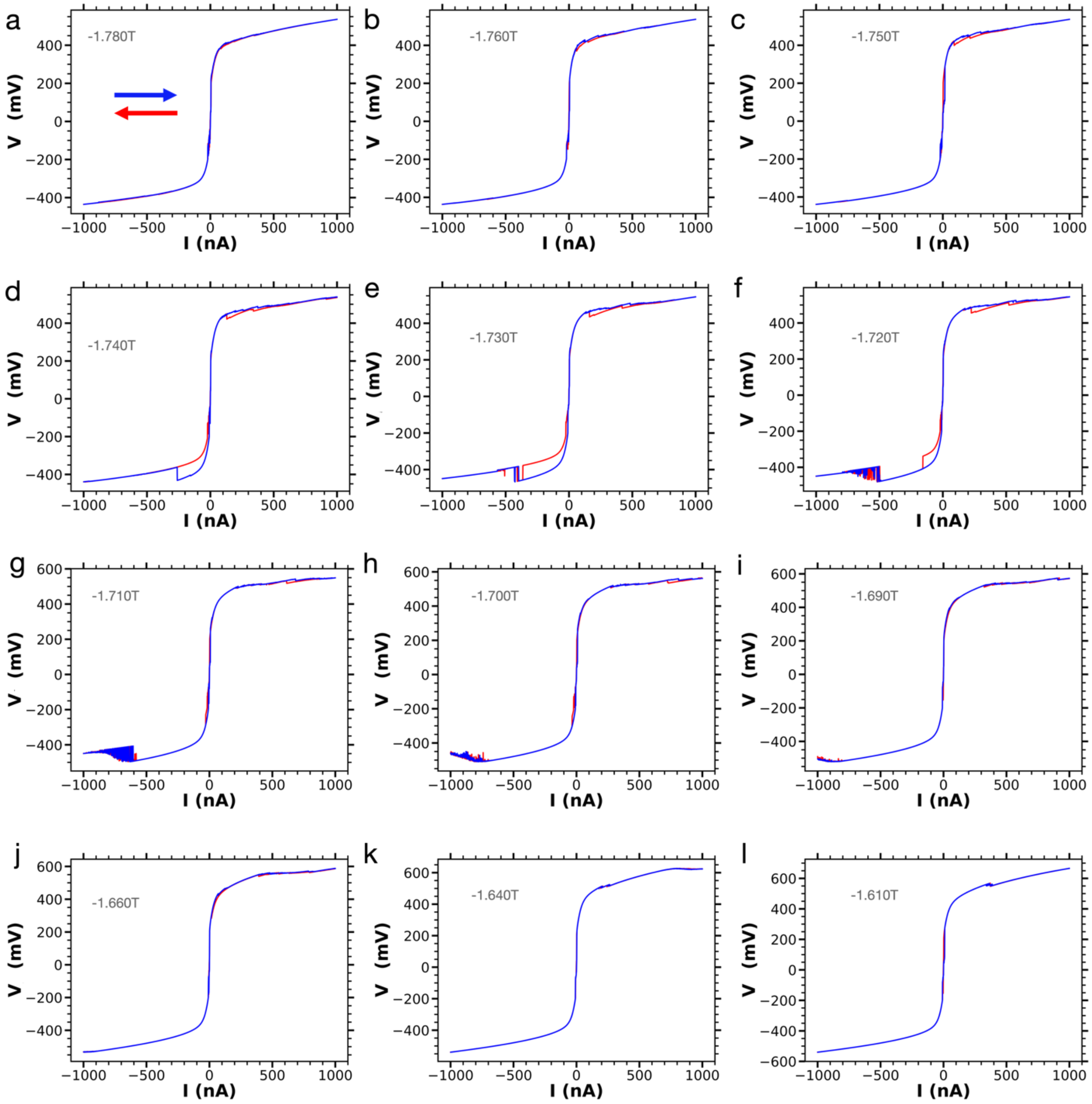


**Fig. S15| Magnetic field dependent *I-V* characteristics for a 4L $CrI_3$. a-l** Voltage as a function of applied current at different magnetic fields near the layer-magnetic phase transition region. All the measurements were performed at $T$ = 1.5 K.

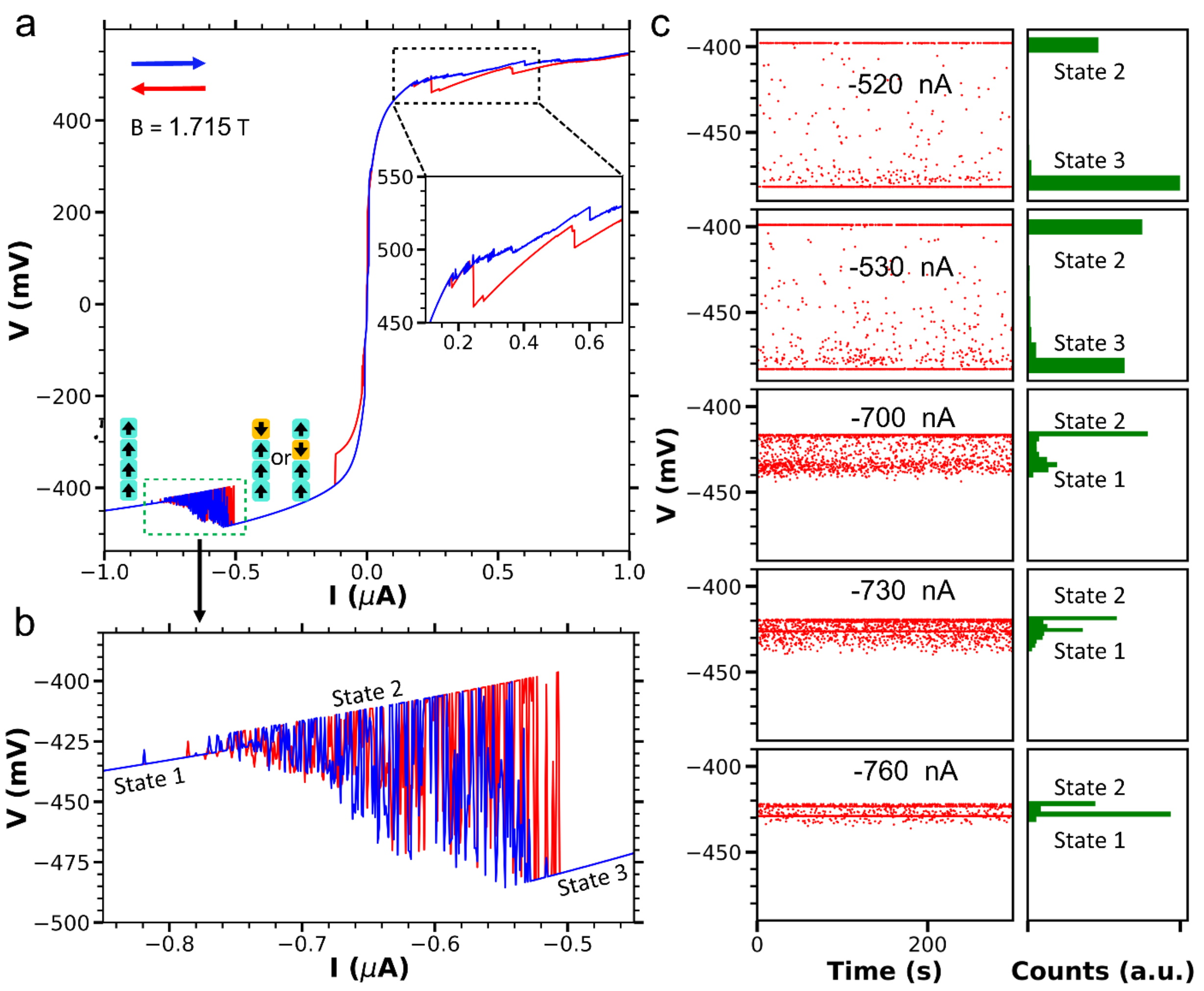


**Fig. S16| Stochastic switching of a four-layer (4L) $CrI_3$. a** Voltage as a function of applied current measured at $B$ = 1.715 T. The inset zoomed from the dashed black square shows the current-driven spin state transition. **b** $V$ versus $I$ curve zoomed from the dashed green square in (a), showing voltage fluctuations between three spin states. **c** Left panels show time snapshots of voltage for constant currents of -760, -730, -700, -530 and -520 nA (from bottom to top). Right panels show the corresponding histograms of voltage distribution with a sampling time of 600 s. All the measurements were performed at $T$ = 1.5 K.

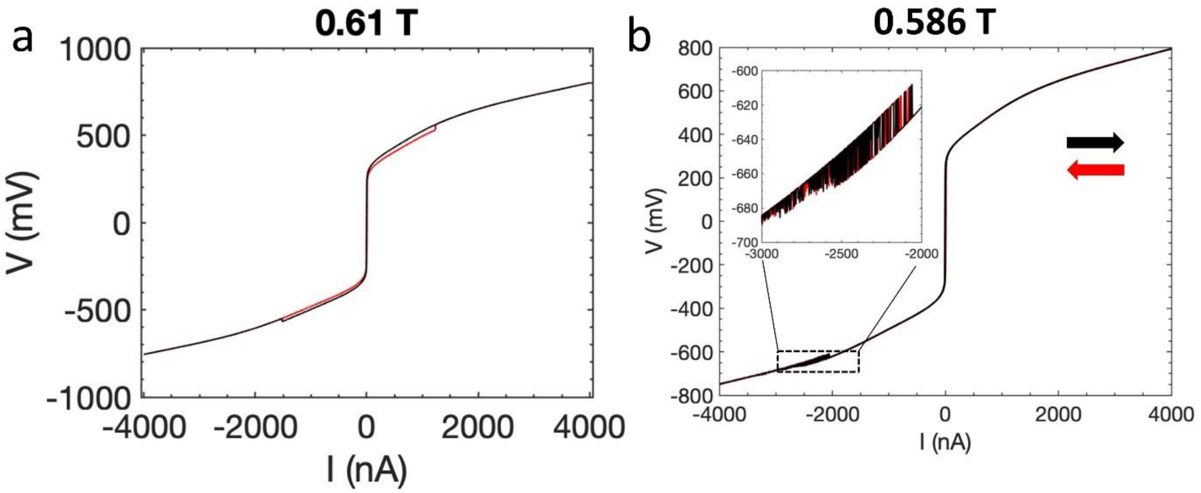


**Fig. S17| Signatures of unidirectional magnetization reversal and stochastic switching in a 5L CrI3 magnetic tunneling device. a** I-V curves were taken at $T$ = 1.5 K, $B$ = 0.61 T and **b** at $T$ = 1.5 K, $B$ = 0.586 T. Black (red) line corresponds to forward (backward) sweeping.

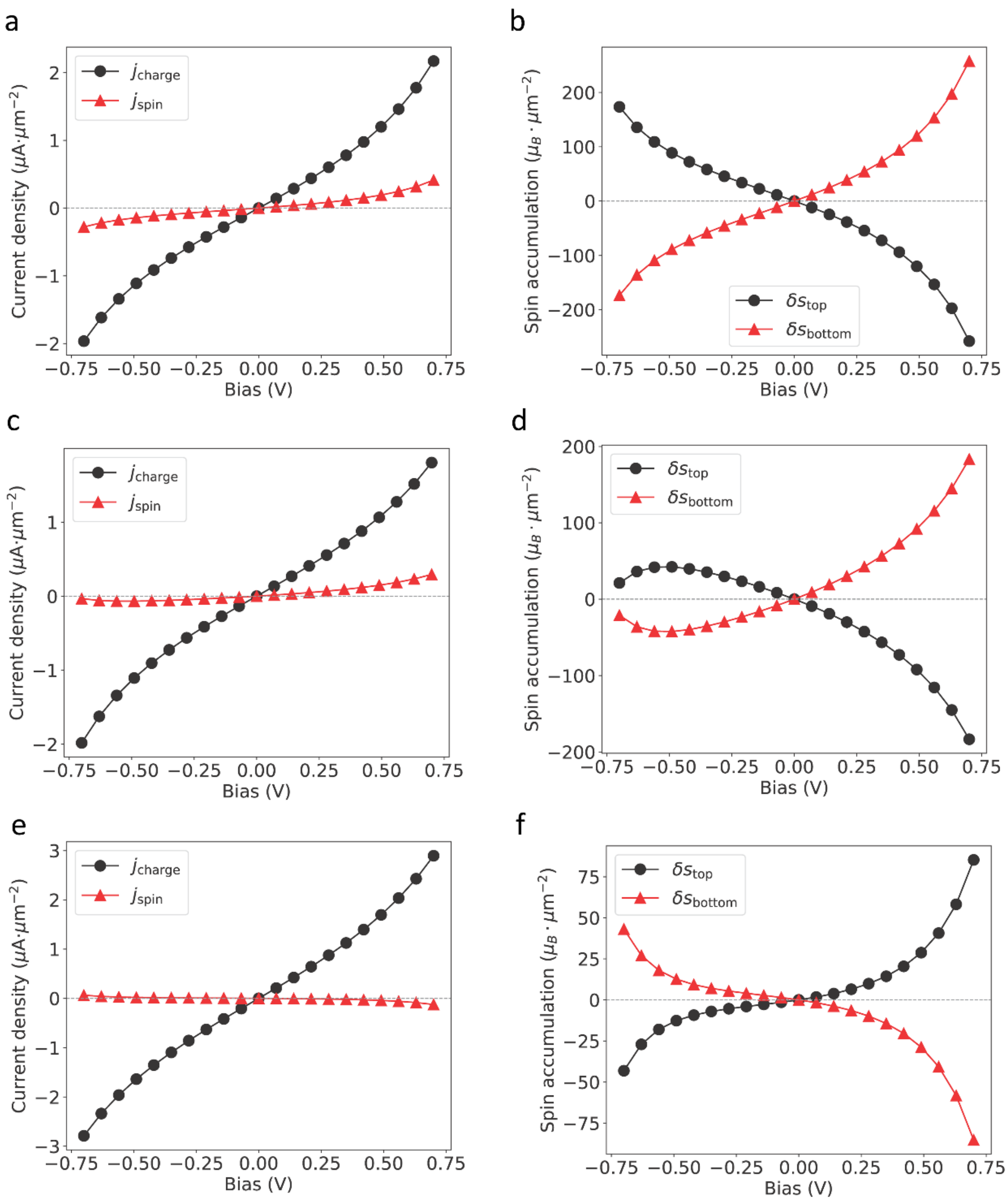


**Fig. S18| Tunneling electron and spin currents (a, c, e) and nonequilibrium spin accumulation (b, d, f) in the SAP with different types of asymmetries. a, b** The top insulator layer ($J_1 < 0$) has a smaller orbital splitting ($\Delta$) than that of the bottom layer by 0.1 eV. **c, d** The bottom insulator layer ($J_2 > 0$) has a higher chemical potential ($\mu_I$) than the top layer by 0.1 eV, mimicking the effect of a perpendicular electric field. **e, f** The hopping between the bottom insulator layer ($J_2 > 0$) and the bottom metal layer is larger than that between the two top layers by 0.01 eV.

**Table. S1| Parameter values used in the NEGF numerical calculation.**

| Parameter | Meaning | Value |
|---|---|---|
| $J$ | Exchange splitting in the insulator layers | 1.4 eV |
| $\Delta$ | Orbital splitting in the insulator layers | $0.8J$ = 1.12 eV |
| $\mu_I$ | Chemical potential of the insulator layers | $-J$ |
| $t$ | Inter-layer hopping | 0.03 eV |
| $W$ | Band width of the metal layers | 2 eV |
| $N_F$ | Fermi-energy density of states for a 1 $\mu$m$^{-2}$ sample | $5 \times 10^4$ eV$^{-1}$ |
| $\Gamma$ | Quasiparticle decay rate due to the leads | 0.003 eV |
| $\tau_s$ | Spin relaxation time in the metal layers | $10^{-10}$ s |

**Table. S2| Summary of $CrI_3$-based tunneling junction devices for tunneling-current-driven magnetic switching and stochastic switching.**

| Device | Temperature | Critical B field * | Critical Current ** | Comments |
|---|---|---|---|---|
| 2L $CrI_3$ No.1 | 1.5 K | ~ 0.500 – 0.592 T | ~ 1000 nA – 5500 nA | |
| 2L $CrI_3$ No.1 | 30 K | ~ 0.520 – 0.580 T | ~ 100 nA – 2000 nA | |
| 2L CrI3 No.2 | 1.5 K | ~ 0.470 – 0.630 T | ~ 5 nA – 100 nA | |
| 4L $CrI_3$ | 1.5 K | ~ 1.60 – 1.80 T | ~ 10 nA – 1000 nA | |
| 5L $CrI_3$ | 1.5 K | ~ 0.56 – 0.65 T | ~ 1000 nA – 4000 nA | |
| 2L $CrI_3$ No.2 | 1.5 K | N/A | N/A | Tunneling junction burnt by Joule heating at ~ 2 $\mu A$ |

* B field for the occurrence of tunneling-current-driven magnetic switching and stochastic switching.
** Magnitude of current bias for the occurrence of tunneling-current-driven magnetic switching and stochastic switching.

## References

[1] S. Datta, "Nanoelectronic Devices: A unified view", arXiv:0809.4460
